# Oil Encapsulation in Core–Shell Filaments from Cellulose Nanocrystals and Methylcellulose via Dry-Jet Wet Spinning

**DOI:** 10.1021/acsomega.6c06865

**Published:** 2026-10-12

**Authors:** Eupidio Scopel, Marc Massicotte, Camila A. Rezende, Emily D. Cranston

**Affiliations:** † Instituto de Química, 28132 Universidade Estadual de Campinas (UNICAMP), P.O. Box 6154, Campinas, São Paulo 13083-970, Brazil; ‡ Department of Wood Science, 8166 University of British Columbia, 2424 Main Mall, Vancouver, British Columbia V6T 1Z4, Canada; § Department of Chemical and Biological Engineering, 8166 University of British Columbia, 2360 East Mall, Vancouver, British Columbia V6T 1Z3, Canada; ∥ UBC Bioproducts Institute, 2385 East Mall, Vancouver British Columbia V6T 1Z4, Canada

## Abstract

The incorporation of oils into structured and water-compatible materials remains a key challenge for cellulose-based materials. Here, we demonstrate the production of core–shell filaments from cellulose nanocrystals (CNCs) and methylcellulose (MC) via a dry-jet wet spinning process, enabling the encapsulation of either plant-based oils or CNC-stabilized Pickering emulsions. The use of a physically entangled MC–CNC hydrogel as the shell allows continuous filament formation from an aqueous formulation without chemical cross-linking. By varying the core composition, distinct structural regimes were obtained: oils alone led to bead-on-string morphologies, while emulsions yielded beadless filaments, consistent with the replacement of a continuous oil core by prestabilized micron-sized oil droplets. Estimated oil contents up to 47 wt % were achieved for corn oil alone and 24 wt % for corn oil emulsions. Turmeric and tea tree oils could also be encapsulated within the MC–CNC shell, although the retained oil content varied with oil type. The modular coaxial spinning setup developed here provides a versatile platform for the fabrication of biobased filaments containing oil. These findings establish a simple strategy to integrate hydrophobic components into continuous cellulose-based materials for future exploration in applications requiring oil-containing structured materials.

## Graphical abstract

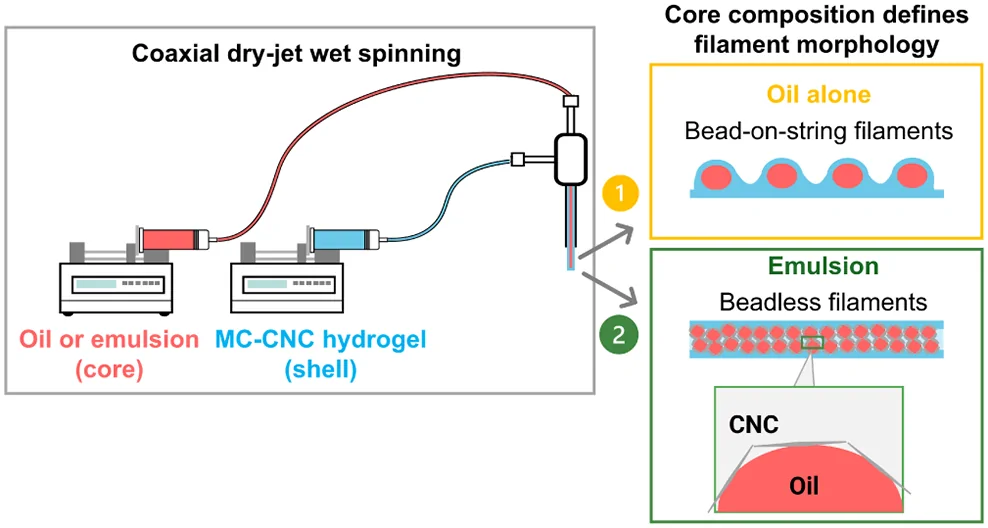

## 1 Introduction

Cellulose has emerged as a promising candidate for the development of sustainable functional materials due to its biodegradability, biocompatibility, and structural versatility.^1–3^ However, the incorporation of hydrophobic components into hydrophilic cellulose matrices remains a major challenge, particularly for the fabrication of continuous and self-supported materials. The intrinsic incompatibility between phases hinders the integration of structural stability with the potential functional properties of hydrophobic compounds. This limitation is critical for applications where matrices are required to contain hydrophobic active agents, including antioxidant, antimicrobial, or therapeutic molecules.^4^ Core–shell architectures where hydrophobic components are spatially confined within a hydrophilic matrix may overcome such challenges while preserving process simplicity.

Wet spinning is a well-established technique for the production of continuous filaments from colloidal dispersions, hydrogels, or polymer solutions, including cellulose solutions or nanocellulose suspensions.^1,5^ In this process, precursors are extruded through a nozzle into a coagulant bath, where they solidify into a continuous filament that can be subsequently collected and dried. When a coaxial nozzle is employed, wet spinning may enable the formation of core–shell filaments, also referred to as core–shell fibers, allowing the encapsulation of a distinct internal phase within a stabilized shell.^6,7^ This configuration provides a promising strategy for a direct route that may overcome the limitations of incorporating hydrophobic components into hydrophilic matrices, enabling the formation of continuous and self-supported materials.

The combination of methylcellulose (MC) and cellulose nanocrystals (CNCs) offers a promising approach for the fabrication of biobased filaments via wet spinning due to their biocompatibility, renewability, and ability to form physically entangled hydrogels.^8–10^ MC is a water-soluble, amphiphilic cellulose derivative widely used as a viscosity modifier,^11–13^ while CNCs are rigid, rod-like nanoparticles obtained from the controlled acid hydrolysis (or oxidation) of cellulose.^14^ MC binds spontaneously and irreversibly to cellulose,^15^ which has enabled the production of highly stable Pickering emulsions,^15^ latexes,^16^ oil powders,^17–19^ gels,^20^ and foams,^21^ as well as low-density paper.^22^ CNCs can contribute as reinforcing agents due to their high stiffness (modulus ∼ 150 GPa), large aspect ratio, and strong hydrogen bonding interactions,^23,24^ while also promoting gelation at lower concentrations and under milder conditions compared to MC or CNCs alone.^20^

MC–CNC hydrogels can be extruded into continuous filaments without the need for chemical cross-linking, facilitating the fabrication of absorbent filaments by wet spinning through relatively simple aqueous processing routes.^25^ By this approach, filaments can be produced without the use of synthetic polymers and energy-intensive drying methods, such as freeze-drying or supercritical drying.^6^ Hynninen et al. demonstrated the production of uniaxial MC–CNC filaments via dry-jet wet spinning, highlighting the importance of MC-to-CNC ratios for spinnability and filament properties.^25^ We believe that this combination of biobased polymer and nanoparticle is particularly attractive for the development of structured materials capable of incorporating hydrophobic components such as oils.

Corn oil is used here as a food-grade demonstration, in place of commonly used hydrocarbon oils such as dodecane and hexadecane.^26^ Plant-based oils also represent attractive hydrophobic components due to their wide availability and intrinsic bioactivity, which often requires encapsulation to preserve anti-inflammatory, antioxidant, and antimicrobial properties.^27,28^ Our platform accommodates a broad range of oils that can function either as the active components (e.g., turmeric or tea tree oils) or as carriers for dissolved or dispersed cosmetic and pharmaceutical ingredients (e.g., corn oil). While oils can be incorporated into aqueous formulations via emulsification and processed into solid oil-containing powders by spray-drying or freeze-drying,^18,26,29,30^ these processing approaches do not form self-supporting structures or standalone shapes.

Core–shell filaments have been demonstrated in high-energy-density and wearable electronics,^7,31,32^ as absorbent^6^ and flame retardant materials,^33^ and conductive cable fibers.^34^ Regarding oil incorporation into cellulose-based structures, Udangawa et al. reported the encapsulation of coconut oil within regenerated cellulose fibers via coaxial electrospinning,^35^ while nanocelluloses have been used in wet-spun filaments containing phase-change materials, particularly as stabilizers for emulsions or capsules.^36–38^ However, to the best of our knowledge, our work is the first demonstration of coaxial wet spinning using a physically entangled MC–CNC hydrogel as the structural shell for encapsulation of liquid oils. This approach offers key advantages, including the use of aqueous formulations, the production of continuous macroscopic filaments, and the independent control of core and shell compositions and flow rates, providing greater flexibility in composition and filament morphology.

Here, we report the encapsulation of oils in continuous core–shell filaments using a physically entangled MC–CNC hydrogel as the shell and either plant-based oils alone or CNC-stabilized Pickering emulsions as the core. The materials were processed using a custom-built dry-jet wet spinning setup equipped with a coaxial nozzle, enabling the formation of distinct filament morphologies. By varying the core composition, distinct structural regimes were obtained, from bead-on-string filaments to beadless structures. The design of the custom-made spinning apparatus and its operation are also detailed, providing a modular approach for continuous filament production. This platform enables the integration of hydrophobic components into structured materials, which may be further explored for applications such as absorbents, wound dressings, fragrance delivery, and edible formulations involving the incorporation or delivery of oils.

## 2 Materials and Methods

### 2.1 Materials

Methylcellulose (MC; Sigma-Aldrich M0262 nominal viscosity grade 400 cP; batch viscosity 425 cP for a 2% aqueous solution at 20 °C; methoxyl content 30.2%), isopropyl alcohol (≥99.5%), 2-[4-(2-hydroxyethyl)­piperazin-1-yl]­ethanesulfonic acid (HEPES buffer), and Nile red were purchased from Sigma-Aldrich. Sodium chloride (NaCl) was purchased from Fisher Chemical. Corn oil was sourced from Mazola (United States), tea tree oil was purchased from Gaia Garden (Canada), and turmeric oil was purchased from Jedwards International (India). Sulfated CNCs were supplied by CelluForce, with an average length of 195 nm and diameter of 5 nm, as measured by atomic force microscopy (AFM) (Supporting Information Section S1, Figure S1), and a sulfate content of 208 mmol/kg, determined by conductometric titration (Supporting Information Section S1).^39^ Additional characterization is available in our benchmarking work.^40^ All materials were used as received without further purification. All water used was Milli-Q grade (Barnstead GenPure Pro, Thermo Fisher Scientific, Waltham, MA) with a resistivity of 18.2 MΩ·cm (referred to as “purified water” throughout).

### 2.2 Fabrication of the Spinning Apparatus

A custom-built wet spinning apparatus was designed to enable coaxial filament production via dry-jet wet spinning. A schematic representation of the filament spinning apparatus is shown in Figure 1. The apparatus comprises two syringe pumps (Fusion 4000-X and Fusion 6000-X, Chemyx), a coaxial nozzle comprising a 22 G inner needle (inner diameter = 0.406 mm, outer diameter = 0.711 mm) and an 18 G outer needle (inner diameter = 0.840 mm, outer diameter = 1.24 mm, Ramehart), a stainless-steel coagulation bath (∼81 cm length, home-built), a height-adjustable T-slot aluminum frame, a custom-built winder with PTFE rods, an infrared (IR) dryer (1500 W Ceramic Space Heater, Brightown), and a submersible conveyor belt (home-built, ∼70 cm useful length) with a variable-speed motor. The setup allows operation in either wet spinning or dry-jet wet spinning mode, depending on the needle position. For dry-jet wet spinning, the needle is positioned above the coagulation bath to create an air gap. Additional construction details, including photographs of the actual setup, mechanical adjustment mechanisms, motor configuration, and bath design, are provided in the Supporting Information (Supporting Information Section S2, Figures S2 and S3).

**Figure 1:**
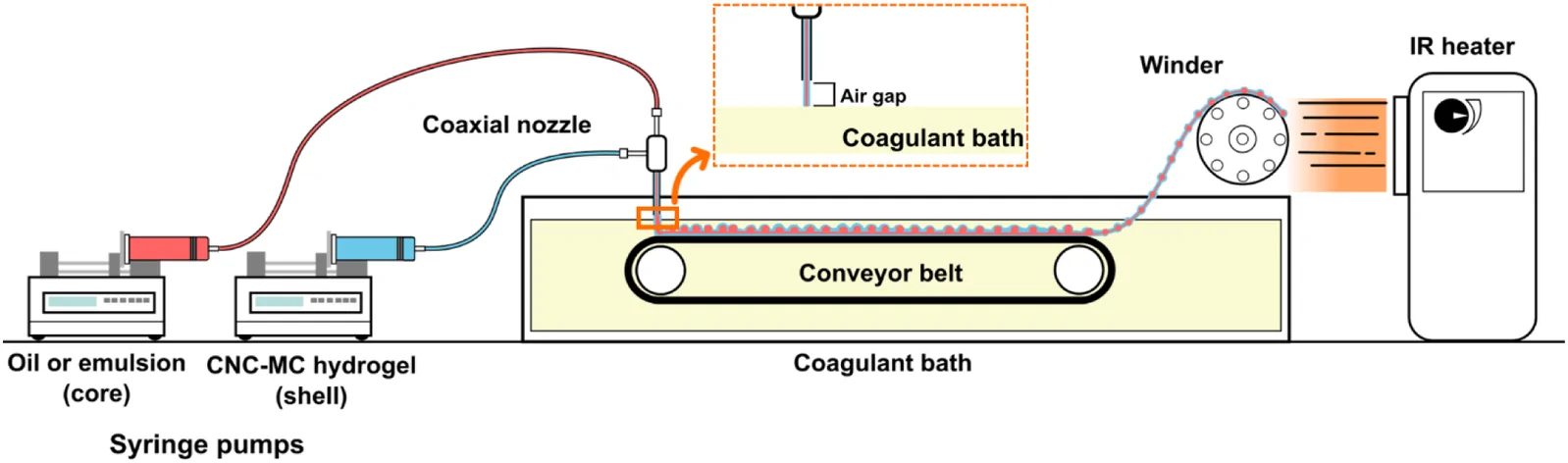
Schematic representation of the dry-jet wet spinning setup, showing the main components and dual feed streams: oil or emulsion (core, in pink) and MC–CNC hydrogel (shell, in blue). The inset (orange box) highlights the air gap required for dry-jet wet-spinning. Note that the schematic is oriented from left to right, whereas the actual setup shown in Figure S2 operates in the opposite direction. The illustrated filament corresponds to the bead-on-string morphology obtained using oil alone as the core.

### 2.3 Preparation of Filaments

Filaments were prepared using a shell composed of MC–CNC hydrogel (1 wt % MC and 2 wt % CNC), following the formulation reported by Hynninen et al.^25^ Either corn oil alone or CNC-stabilized Pickering emulsions were used as the core component.

The MC–CNC hydrogel was prepared by dispersing MC powder into an aqueous CNC suspension to reach the target concentrations. The mixture was heated to 85 °C, stirred for 30 min, and subsequently cooled to 4 °C overnight. Then, it was stirred for 24 h at room temperature and stored at 4 °C until use. Before extrusion, the hydrogel was transferred to a Pyrex crystallizing dish, and bubbles were removed under vacuum (Cole Parmer HWS 6460A) for 5 h. This step was critical to ensure successful filament formation, as it reduced bubble content more effectively than centrifugation, particularly given the high viscosity of the hydrogel.

Pickering emulsions were prepared following the protocol by Massicotte and Cranston,^18^ with minor modifications. Emulsions were formulated using corn oil at a 20:80 oil-to-water volume ratio. The aqueous phase contained 0.25 wt % CNCs, 50 mM NaCl, and 25 mM HEPES buffer. CNC suspensions were first mixed with the NaCl solution in a beaker under vigorous magnetic stirring, followed by the addition of the HEPES buffer. Corn oil was then added dropwise into the center of the vortex, and the mixture (total volume: 30 mL) was transferred to a centrifuge tube. A pre-emulsion was formed by vortexing the tube for 30 s, followed by sonication (Branson 550) at 60% amplitude for 20 s in an ice bath (1 s on/1 s off). This vortex-sonication cycle was repeated five times.

For filament fabrication, approximately 20 mL of the MC–CNC hydrogel was carefully transferred into a 20 mL stainless steel syringe (Chemyx) to reduce air incorporation and connected to the spinning setup. Similarly, approximately 20 mL of either oil alone or Pickering emulsion was placed in a 50 mL plastic syringe and connected to the coaxial nozzle. An additional filament was prepared by extruding only the MC–CNC hydrogel shell, without any oil or emulsion as the core. Due to the high viscosity of the MC–CNC hydrogel, a high-pressure syringe pump (Fusion 6000-X) was used to control the flow, while a second pump (Fusion 4000-X) was used for the lower-viscosity core phase. The extrusion flow rates were set to 0.5 mL min^–1^ for the shell and 0.1 mL min^–1^ for the core.

Filaments were extruded through the coaxial nozzle into the coagulant bath containing only isopropyl alcohol, which was selected as the coagulation medium based on previous wet spinning of hydrogels using alcohols.^25,41^ The nozzle was placed ∼0.5 cm above the surface of the coagulant to create an air gap, which was kept fixed throughout the experiments, enabling a dry-jet wet spinning configuration, as illustrated in Figure 1 (inset). This configuration was essential to prevent premature coagulation, which occurred when the nozzle was submerged in the coagulation bath. In such conditions, the buoyancy of the encapsulated oil caused the filaments to float and rupture at the surface, preventing continuous extrusion.^42^ In contrast, the dry-jet configuration maintained structural integrity during spinning. The coagulation conditions were kept fixed, and filaments were allowed to coagulate along the full length of the bath, then collected using the rotating winder. The conveyor belt speed was manually adjusted to approximately 1 cm s^–1^ to allow continuous filament collection without visible stretching and was not systematically varied. At least five independent trials were performed for each formulation. Spinning was intentionally stopped after sufficient material had been collected (typically 60 cm to 1 m of filament produced per run). The collected filaments were continuously dried with an infrared heater for 10 min.

### 2.4 Characterization

Rheological measurements were performed using a rotational rheometer (Kinexus Ultra+, Malvern Panalytical) equipped with a cone–plate geometry (40 mm, 4°). Measurements were conducted at 25 °C and in duplicate (representative curves are shown). Flow curves were obtained by measuring apparent viscosity as a function of shear rate (from 1 to 500 s^–1^) for the MC–CNC hydrogel, CNC-stabilized Pickering emulsion, and corn oil. The flow curves were fitted using the power-law model (eq 1)­

1 $$\eta =K{\mathrm{\gamma ̇}}^{n-1}$$

where *η* is the apparent viscosity, K is the consistency index, γ̇ is the shear rate, and n is the flow behavior index.

To relate the flow curves to the extrusion conditions, the nominal shear rate in the inner needle was estimated from the core flow rate and nozzle diameter (Supporting Information, Section S3).^43^ Oscillatory amplitude sweep tests were performed for the MC–CNC hydrogel and CNC-stabilized Pickering emulsion at a fixed frequency of 1 Hz over a strain range of 1–800% to evaluate the viscoelastic behavior of the formulations. Storage (G′) and loss (G″) moduli were used to characterize the linear viscoelastic response and G′–G″ crossover.

Emulsions and filaments were analyzed by polarized optical microscopy (POM, Nikon Eclipse LV100N POL) equipped with a Nikon DS-Ri2 camera (Nikon Instruments) under crossed polarizers. Emulsion droplet size and filament thickness were measured using ImageJ software. For droplet-size distributions, 100 droplets from at least two distinct sample areas were measured for each condition.

Thermogravimetric analysis (TGA) was performed using a TGA 5500 (TA Instruments) at a heating rate of 10 °C min^–1^ from room temperature to 600 °C under nitrogen atmosphere. Oil content in the filaments was estimated from TGA curves in duplicate using the temperature interval corresponding to the degradation of oils as a reference. The mass loss within this range was attributed to the encapsulated oil. MC–CNC was analyzed as a reference to assess overlaps between the thermal responses of the shell and the oils. Because these thermal events are not completely separated, the resulting values are considered estimates of oil content rather than absolute measurements. Oil loading per unit length was calculated by dividing the estimated oil mass by the corresponding filament length.

## 3 Results and Discussion

### 3.1 Rheological Behavior of Shell and Core Formulations

Rheological measurements were used to compare the flow behavior of the MC–CNC hydrogel, CNC-stabilized Pickering emulsion, and corn oil, as well as the viscoelastic behavior of the MC–CNC hydrogel and Pickering emulsion (Figure 2). Both the MC–CNC hydrogel and Pickering emulsion exhibited shear-thinning behavior, while corn oil showed an approximately Newtonian response over the investigated shear-rate range, as expected (Figure 2a). Power-law fitting yielded flow behavior indices (n) of 0.05 and 0.24 for the MC–CNC hydrogel and Pickering emulsion, respectively, while n was approximately 1 for corn oil (Table S1).

**Figure 2:**
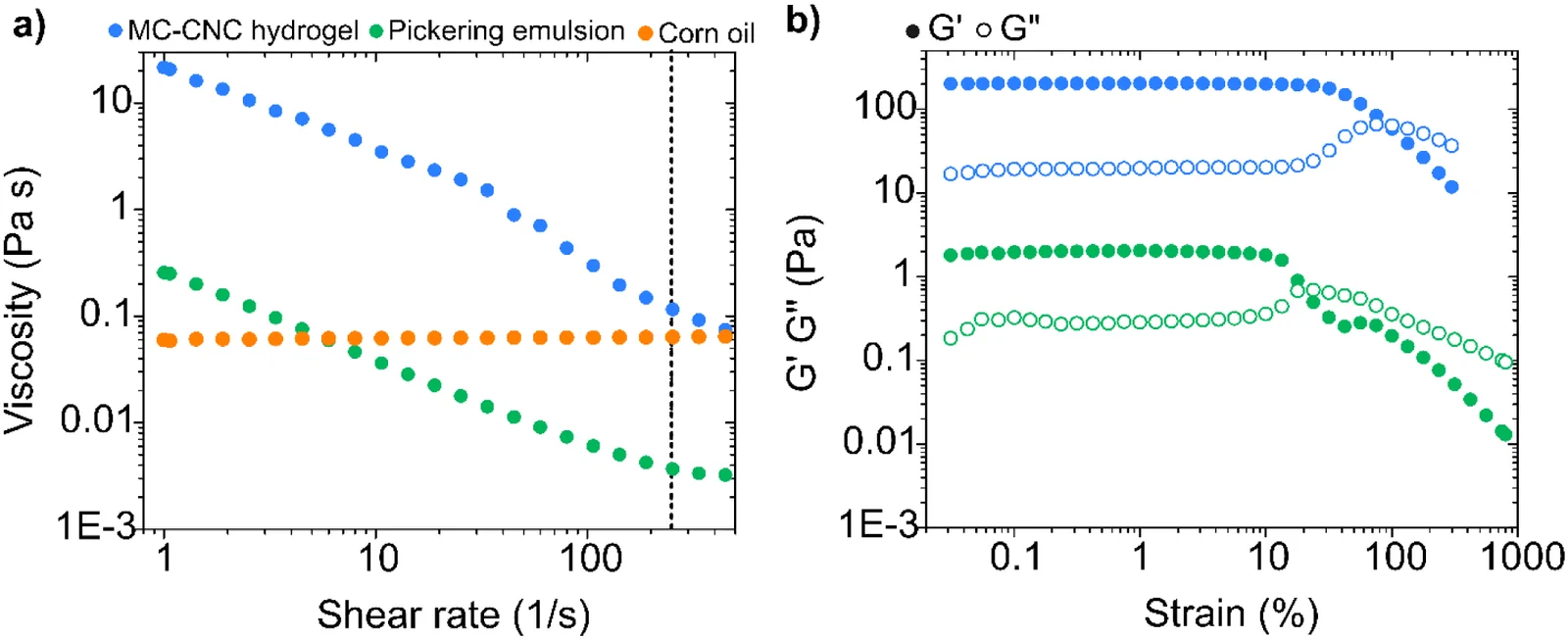
Rheological behavior of the shell and core formulations: a) viscosity as a function of shear rate, where the dashed line indicates the estimated nominal shear rate in the inner needle during core extrusion; b) oscillatory amplitude sweeps showing storage (G′) and loss (G″) moduli for the MC–CNC hydrogel and Pickering emulsion as a function of strain.

To relate the flow curves to the conditions experienced by the core during extrusion, the nominal shear rate in the inner needle was estimated from the core flow rate (0.1 mL min^–1^) and the inner needle diameter (0.406 mm), resulting in approximately 250 s^–1^ (details provided in Supporting Information Section S3). At this shear rate, indicated with a dashed line in Figure 2a, the Pickering emulsion exhibited a lower viscosity than corn oil (0.004 versus 0.06 Pa·s, respectively). Thus, although the Pickering emulsion is more viscous than corn oil at low shear rates (Figure 2a), its pronounced shear-thinning behavior results in a lower viscosity at shear rates around those expected during extrusion.

Oscillatory amplitude sweep experiments demonstrated the distinct viscoelastic behavior of the MC–CNC hydrogel and Pickering emulsion (Figure 2b). Corn oil was not included in the amplitude sweep analysis because of its predominantly Newtonian liquid behavior. Within the linear viscoelastic region, the MC–CNC hydrogel exhibited a predominantly elastic response, with G′ substantially exceeding G″ (G′ ∼ 200 Pa and G″ ∼ 20 Pa). The Pickering emulsion also exhibited G′ > G″ at low strain, although its moduli were approximately 2 orders of magnitude lower (G′ ∼ 2 Pa and G″ ∼ 0.3 Pa). The G′–G″ crossover, associated with the transition from predominantly elastic to viscous behavior, occurred at approximately 94% strain for the MC–CNC and 21% for the Pickering emulsion, indicating that the MC–CNC network remained predominantly elastic up to higher strains. Overall, the rheology results highlight the markedly different behavior of the shell and core formulations under the conditions investigated, providing context for the processing during wet spinning.

### 3.2 Filaments Prepared Using Oil Alone as the Internal Component

The MC–CNC hydrogel was suitable as a shell material for coaxial dry-jet wet spinning, as demonstrated in Figure 3. As expected, Figure 3a shows that shell-only MC–CNC filaments, prepared without any oil or emulsion core, were long and continuous under the evaluated conditions.^25^ MC is known to adsorb onto CNC surfaces when mixed,^15,20^ resulting in synergistic interactions that promote hydrogel formation and processability. In this hydrogel, CNCs act as rigid elements that physically cross-link and reinforce the MC network and enable partial structural recovery after extrusion.^25^ As discussed in Section 3.1, the MC–CNC formulation forms a predominantly elastic, shear-thinning hydrogel suitable for extrusion-based processing, in which the high viscosity before extrusion is substantially reduced when shear is applied, allowing easier flow.

**Figure 3:**
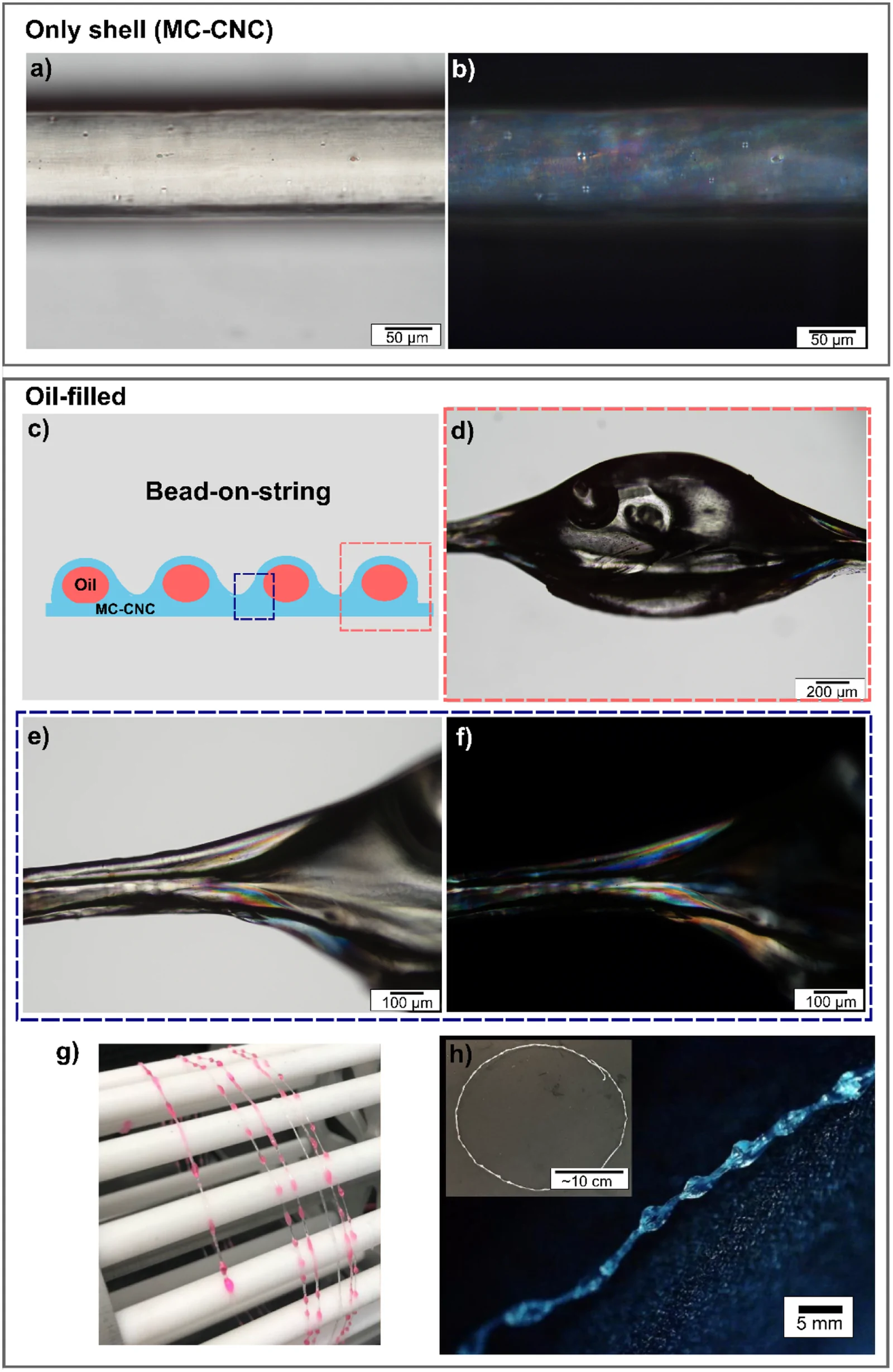
Morphology of wet-spun MC–CNC-based filaments: a) MC–CNC filament (shell only) under optical microscopy and b) with polarized light; c) schematic representation of the bead-on-string morphology of MC–CNC filaments with corn oil as the core; d) oil-rich region and e) neck region under optical microscopy; f) the same region as e) with polarized light; g) photograph of a tea tree oil-filled filament being collected in the winder, where the oil is stained with Nile Red to facilitate visualization; and h) macroscopic view of a tea tree oil-filled filament after drying and collection.

The POM image in Figure 3b shows slight birefringence, indicating anisotropic ordering within the MC–CNC filament. However, the origin of this birefringence cannot be explicitly attributed to flow-induced CNC alignment during spinning since CNCs can develop local ordered domains under semidilute conditions,^44^ and interactions with adsorbing polysaccharides can increase their effective volume fraction and promote anisotropic domains.^45^ MC–CNC hydrogels have also previously been reported to exhibit birefringence.^20^ As shear and extensional flows during extrusion are known to promote CNC orientation,^46^ dry-jet wet spinning may further orient preexisting anisotropic structures. Occasional localized bright regions are also observed, suggesting local variations in birefringence within the filament structure.

Despite the synergistic interactions between MC and CNCs, successful filament formation requires careful control of component ratios. The conditions adopted here (1 wt % MC and 2 wt % CNCs) follow the previous work from Hynninen et al.,^25^ which identified an optimal compositional window for wet spinning of uniaxial MC–CNC filaments. Under these conditions, there is sufficient network formation that enables gelation while maintaining a rheological profile suitable for extrusion. Notably, neither MC nor CNC alone at such low concentrations can form extrudable filaments. For example, uniaxial CNC-only filaments have been reported only from concentrations above 10 wt %, where CNC dispersions exhibit gel-like behavior.^47^ More broadly, CNCs have been successfully combined with different polymer matrices using dry-jet wet spinning, including poly­(ether sulfone),^48^ poly­(butylene adipate-*co*-terephthalate),^49^ and poly­(ether imide).^50^ As a baseline characterization of the shell material, uniaxial tensile testing was performed on dried shell-only MC–CNC filaments. The filaments exhibited stress at break of 9 ± 3 MPa, strain at break of 2.8 ± 0.8%, and Young’s modulus of 0.9 ± 0.3 GPa (n = 9, Figure S4).

Corn oil was successfully incorporated as the internal component using the coaxial nozzle, resulting in bead-on-string filaments (Figure 3c–f). This morphology consists of alternating oil-rich “beads” and oil-depleted “necks”, which is consistent with a capillary instability of the confined continuous oil core within the viscoelastic MC–CNC shell. The continuous oil thread formed during extrusion can break up into more spherical oil-rich domains, reducing the interfacial area and associated free energy. This behavior observed during filament formation (Supporting Video 1) is consistent with Rayleigh–Plateau-type instability. It is interesting to note that the resulting filaments are not perfectly cylindrical, as slight deformation and partial flattening occur during drying, particularly in the neck regions and occasional air inclusions may remain in the filament despite vacuum degassing of the highly viscous MC–CNC hydrogel, which could potentially affect mechanical properties of the filaments.

The MC–CNC hydrogel maintained its structural integrity despite the localized deformation induced by the encapsulated oil, enabling core–shell filament formation under the investigated conditions. In the oil-filled filaments, beads reached up to 1000 μm in height, while the neck regions ranged from 130 to 180 μm in thickness. In comparison, shell-only MC–CNC filaments exhibited thickness of approximately 80 μm. For reference, the outer nozzle inner diameter is 840 μm, while the inner needle outer diameter is 711 μm.

The oil-filled filaments also exhibited birefringence under polarized light (Figure 3f), indicating that anisotropic ordering was retained in the bead-on-string morphology. Additional trials showed that when the oil extrusion rate was increased to 0.25 mL min^–1^, the MC–CNC shell ruptured, likely due to the formation of excessively large beads that compromised the shell integrity, indicating that an adequate balance between extrusion rates is required to enable structure formation. In addition, turmeric and tea tree oils were also successfully encapsulated under the same processing conditions, also exhibiting bead-on-string morphologies, which demonstrates the versatility of the proposed platform across different oils. The ability to produce long and continuous filaments that can be collected is demonstrated in Figure 3g, where tea tree oil-filled filaments are shown during collection on the winder. These filaments could be continuously collected over extended lengths and handled after processing. A macroscopic view of the dried filaments is shown in Figure 3h.

To the best of our knowledge, this is the first report using a physically entangled MC–CNC hydrogel as the structural shell for the encapsulation of liquid oils by coaxial dry-jet wet spinning. Although bead-on-string morphologies have been reported in electrospun materials containing cellulose,^35^ the underlying mechanisms and application spaces differ significantly. Electrospun fibers are typically submicrometers in diameter, rely on high electric fields, and often require volatile solvents. In contrast, wet spinning enables the production of continuous, macroscale filaments from aqueous formulations. In addition, the independent core and shell feed streams allow the internal phase to be modified without changing the structural MC–CNC shell, as demonstrated here by the use of oils and Pickering emulsions. For this reason, potential applications of these filaments may include absorbent materials, wound dressings, and controlled-release devices.

### 3.3 Filaments Prepared Using Pickering Emulsions as the Internal Component

Core–shell filaments were also produced using CNC-stabilized corn-oil-in-water Pickering emulsions (20:80 by volume) as the internal component. In contrast to the oil-alone core, which forms a continuous internal phase, the Pickering emulsion contains prestabilized micron-sized oil droplets dispersed in water, replacing the continuous oil thread susceptible to capillary breakup.

Figure 4 shows an optical microscopy image of the Pickering emulsion along with the corresponding droplet size distribution. Prior to filament preparation, the CNC-stabilized Pickering emulsions exhibited an average droplet diameter of 7 ± 4 μm and remained stable for over 6 months, with no observable oil leakage. This stability is attributed to the adsorption of CNCs at the oil–water interface, which prevents coalescence by imparting some electrostatic repulsion and forming a physical barrier around droplets.^26^ Extrusion under the same conditions used for filament spinning did not visibly destabilize the emulsion, as no phase separation was observed and the average droplet diameter remained similar after processing (7 ± 3 μm; Figure S5). Compared to emulsions stabilized by traditional surfactants, Pickering emulsions typically show greater resistance to coalescence and long-term stability.^51^ When stabilized with CNCs, they are also renewable and biobased unlike most oil-derived surfactants.^26^

**Figure 4:**
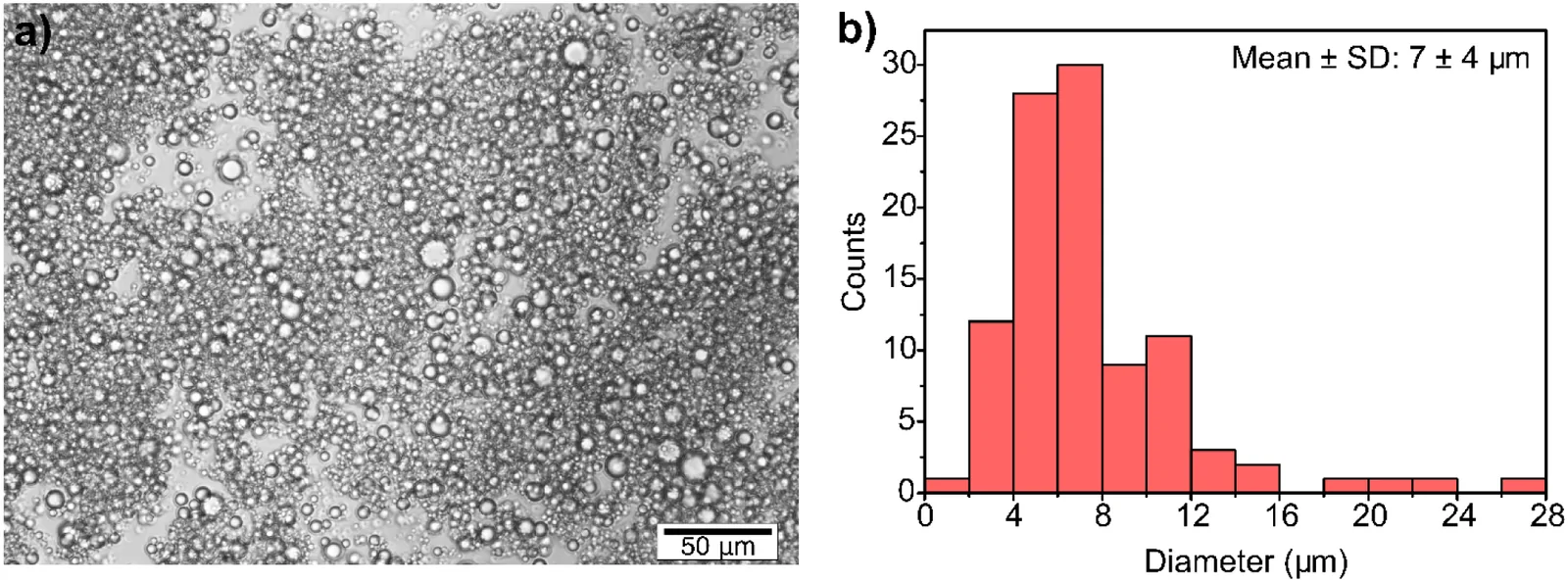
a) Optical microscopy of a CNC-stabilized corn-oil-in-water Pickering emulsion and b) oil droplet diameter distribution histogram. Similar results were observed after extrusion of the emulsion through the coaxial nozzle used for filament spinning (Figure S5).

Filaments with CNC-stabilized emulsions as the core exhibited a uniform, beadless morphology (Figure 5), with a filament thickness ranging from 300 to 350 μm along their length. The absence of a continuous oil thread in the emulsion core limits the formation of large oil domains susceptible to capillary-driven breakup, providing a plausible explanation for the more uniform morphology compared to the bead-on-string structures obtained with oil alone (Figure 3). At the estimated shear rate during core extrusion (∼250 s^–1^), the Pickering emulsion exhibited lower viscosity than corn oil (Section 3.1), which may additionally influence flow behavior during spinning.

**Figure 5:**
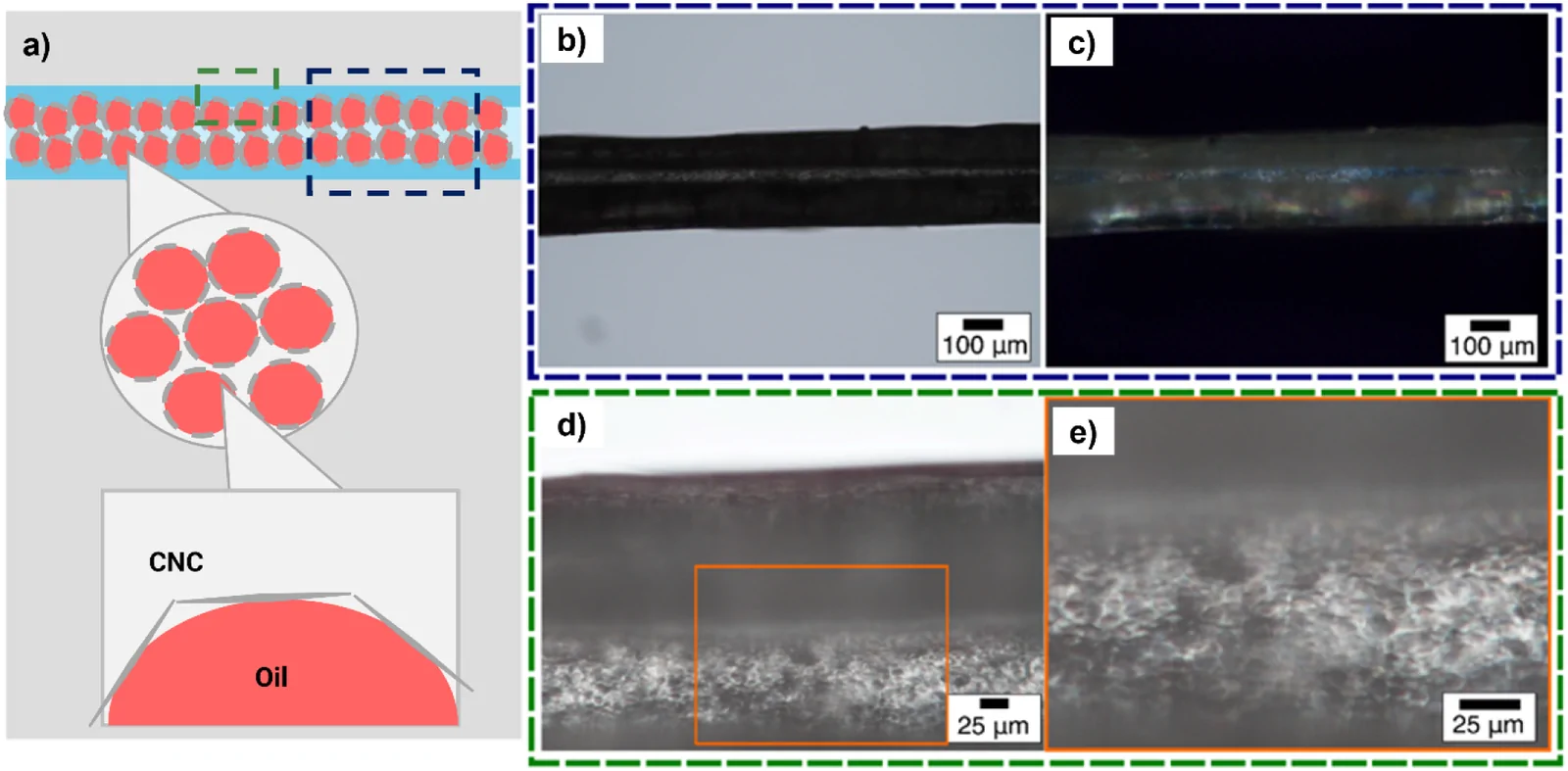
Morphology of Pickering emulsion-filled filaments: a) schematic representation of the CNC-stabilized Pickering emulsion used as the core phase, showing oil droplets dispersed and CNCs at the oil interface; b) filament under optical microscopy and c) with polarized light; d–e) close-up microscopy images highlighting the internal droplet-rich domains within the filament core.

Close-up images of the filaments (Figure 5d–e) reveal quasi-spherical structures embedded within the filament core. These features are consistent with oil droplets, suggesting that the droplet organization is at least partially preserved during coagulation and drying. However, the dried filaments should no longer be considered a bulk emulsion, as coagulation and drying are expected to remove the aqueous phase, leading to a compact structure composed of CNC-stabilized oil droplets embedded within the MC–CNC matrix. Such structures are absent in filaments composed solely of the MC–CNC shell (Figure 3a). Similar to both oil-filled and shell-only filaments, the emulsion-filled filaments also exhibited birefringence under polarized optical microscopy (Figure 5c).

### 3.4 Oil Content and Outlook for Coaxial Filaments

TGA was used to estimate the oil content in the core–shell filaments using the degradation interval of oil alone as a reference (Figure 6a).^18^ MC–CNC was analyzed to assess its contribution to the thermal response. The main degradation of the MC–CNC occurred between 280 and 340 °C, with a maximum degradation rate near 317 °C, followed by a slower mass-loss region at higher temperatures. Corn oil exhibited mass loss between 362 and 477 °C, which partially overlaps with the high-temperature mass loss of the shell. Accordingly, the mass loss of the filaments within this range was attributed to the encapsulated oil, and the TGA-derived values are therefore considered estimates of oil content. Filaments encapsulating corn oil alone exhibited an average oil content of 47 ± 6 wt %, whereas emulsion-filled filaments exhibited a lower oil content of 24 ± 4 wt % (Figure 6b). When normalized by filament length, oil-filled filaments encapsulate approximately 0.4 mg of oil per cm, compared to 0.1 mg cm^–1^ for emulsion-filled filaments. This difference is expected based on the composition of the core phase, as the Pickering emulsion contains only 20% oil by volume, inherently limiting the maximum achievable loading, even though emulsions are evenly distributed along the filament length. For both filaments, only small mass losses were observed below 100 °C, suggesting a low amount of residual moisture in the dried filaments.

**Figure 6:**
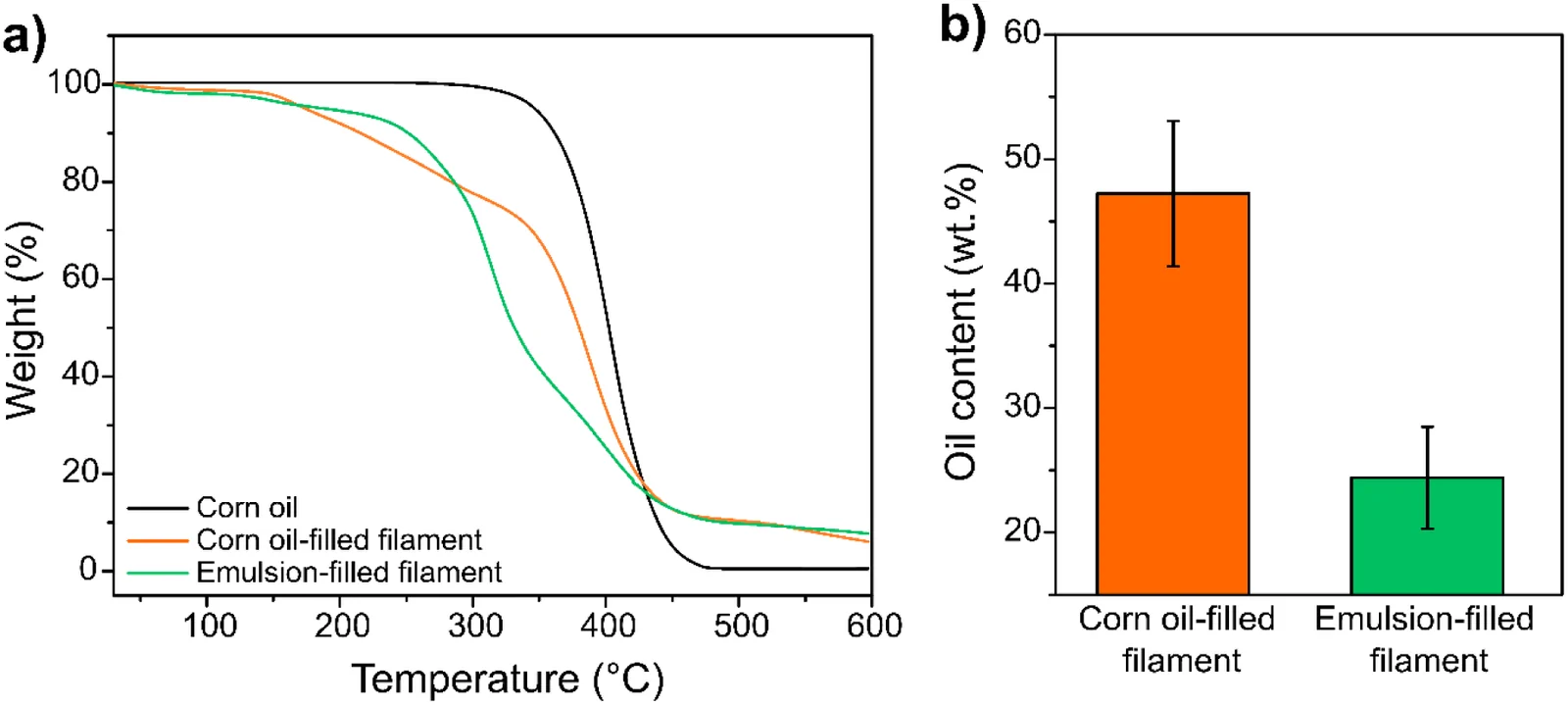
TGA and estimated oil content of core–shell filaments: a) TGA curves of corn oil, oil-filled filaments, and emulsion-filled filaments; b) quantification of estimated encapsulated oil based on TGA.

In comparison, turmeric oil-filled filaments contained 35 wt % oil, while tea tree oil-filled filaments contained 5 wt % oil (Figure S6 and Table S2). The lower oil content observed for tea tree oil is consistent with its higher volatility, which is also reflected in its lower-temperature mass-loss profile during TGA. However, other oil-specific properties may also contribute to the observed differences in oil retention. In particular, oil polarity is known to influence CNC adsorption and interfacial behavior at oil–water interfaces,^52,53^ and differences in affinity with the MC–CNC shell may also play a role. It is important to highlight that the TGA measurements represent the amount of oil retained in the dried filaments at the time of analysis. For more volatile oils, some oil may be lost during drying, while gradual release during storage may also occur for less volatile oils. Since oil retention was not monitored over time or under different storage conditions, the long-term retention remains to be evaluated. Indeed, the characteristic odor of tea tree and turmeric oils remained noticeable after drying.

The versatility of the proposed spinning platform is demonstrated by its ability to accommodate different core components and generate distinct filament morphologies. Bead-on-string filaments may provide localized oil reservoirs, while uniform beadless filaments may be of interest for future controlled release applications because of the more distributed oil along the filament length. These morphologies may be advantageous for different future applications, which remain to be systematically evaluated. For example, mechanical deformation can induce structural rupture and promote oil release (Figure S7), suggesting a possible route for content delivery. These characteristics may motivate future evaluation of the filaments in applications involving bioactive oils with antimicrobial or anti-inflammatory properties, which could be explored in advanced wound dressings as alternatives to conventional additives such as silver nanoparticles.^54^ Similarly, volatile oils may be considered for fragrance delivery, while structured filaments could be adapted for food-related applications, involving oil-based nutrients or functional lipids. In addition, the incorporation of phase change materials^35^ could also be considered for applications requiring thermal regulation in textiles or packaging. However, application-specific performance, including release kinetics, mechanical properties, and biological or functional activity, remains to be evaluated.

Finally, the modular spinning apparatus developed in this work can be readily adapted to different geometries or compositions. Beyond core–shell filaments, simple modifications, such as replacing the rotating winder with a flat collector, could enable the production of nonwoven mats, further expanding the application scope. This apparatus can also be extended to other physically cross-linked hydrogels, offering a versatile and biobased alternative for the fabrication of functional materials.^6^

## 4 Conclusion

In this work, we incorporated either plant-based oils or CNC-stabilized emulsions as the internal phase of core–shell filaments within an MC–CNC shell. The morphology of the resulting filaments was strongly dependent on the nature of the core phase: bead-on-string filaments were obtained with oil alone, consistent with capillary-driven breakup of the continuous oil core. On the other hand, emulsion-filled filaments exhibited uniform and continuous morphologies, consistent with the replacement of the continuous oil core by prestabilized micron-sized oil droplets dispersed in water. These distinct structures demonstrate the tunability of the platform and may be relevant for future applications requiring different oil distributions within continuous materials. By combining physically assembled hydrogels, coaxial wet spinning, and flexibility in core composition, this approach enables the fabrication of continuous cellulose-based filaments containing hydrophobic components. Overall, these results broaden the structural and compositional possibilities of wet-spun cellulose materials for oil incorporation.

## Supplementary Material





## Data Availability

Data will be available on request.

## Notes

Section S1: CNC characterization; Section S2: additional wet spinning information; Section S3: rheological data analysis; Section S4: mechanical properties of shell-only MC-CNC filaments; Section S5: additional Pickering emulsion characterization; Section S6: additional oil-filled filament characterization (PDF)
Supporting Video 1. Bead formation during coaxial dry-jet wet spinning of oil-filled MC–CNC filaments (MP4)
