# Supplementary material for: Oil Encapsulation in Core–Shell Filaments from Cellulose Nanocrystals and Methylcellulose via Dry-Jet Wet Spinning
Source: ACS Omega. 2026 Oct 12;11(42):65045–54. doi: 10.1021/acsomega.6c06865 (PMC13718204; doi:10.1021/acsomega.6c06865)
Supplement: Supplementary file 1 [file ao6c06865_si_001.pdf]

Supporting Information

Oil encapsulation in core-shell filaments from cellulose nanocrystals and methylcellulose via dry-jet wet spinning

Eupidio Scopel,[a,b] Marc Massicotte,[c] Camila A. Rezende,[a] Emily D. Cranston[b,c,d*]*

[a] Universidade Estadual de Campinas – UNICAMP, Instituto de Química, 13083-970, Campinas, SP, Brazil.

[b] Department of Wood Science, University of British Columbia, 2424 Main Mall, Vancouver, BC V6T 1Z4, Canada

[c] Department of Chemical and Biological Engineering, University of British Columbia, 2360 East Mall, Vancouver, BC V6T 1Z3, Canada

[d] UBC Bioproducts Institute, 2385 East Mall, Vancouver BC, V6T 1Z4, Canada

* Corresponding authors

Table of Contents

S1. CNC characterization 2
S2. Additional wet spinning information 3
S3. Rheological data analysis 6
S4. Mechanical properties of shell-only MC-CNC filaments 7
S5. Additional Pickering emulsion characterization 8
S6. Additional oil-filled filament characterization 9

S1. CNC characterization

The morphology of cellulose nanocrystals (CNCs) was analyzed by AFM using a FlexAFM equipped with a C3000 controller (Nanosurf), operating in non-contact mode with an NCHR probe (PointProbe Silicon SPM Sensor, Nanoworld – tip radius: 8 nm; cantilever resonance frequency: 320 kHz, nominal force constant: 42 N m^{-1}; thickness: 4 μm). Prior to imaging, CNC dispersions were diluted to 5 μg mL^{-1}, deposited onto freshly cleaved mica substrates, and dried at room temperature in a desiccator. Topography images were processed using Gwyddion 2.56 (Gwyddion.net). A total of 100 particles were measured to obtain diameter and length histograms. CNC diameters were determined from height profiles.

The sulfate content was determined by conductometric titration of 0.2 g of CNCs (dry basis) suspended in 200 mL of MilliQ and 2 mL of 0.1 M NaCl. The sulfate content was determined from the strong acid equivalence point.

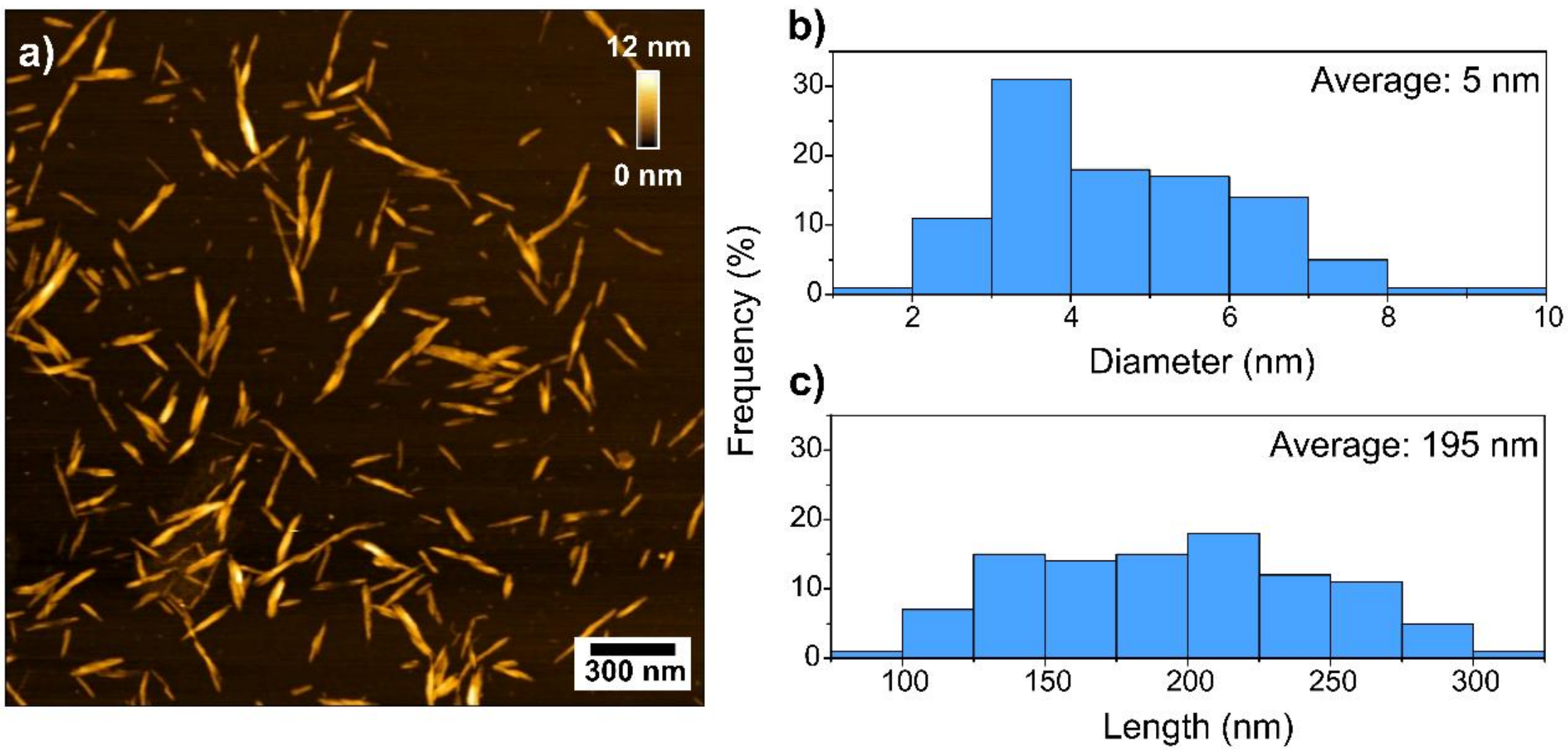

Figure S1. Characterization of CNCs: (a) AFM topography image; (b) diameter distribution histogram; and (c) length distribution histogram. The calculated aspect ratio (L/D) is 47.

S2. Additional wet spinning information

A custom-built wet spinning setup was developed to enable the production of coaxial filaments under both dry-jet wet spinning conditions and standard wet spinning conditions. Photographs of the apparatus are provided in Supplementary Figures S2 and S3.

The system consists of the following components:

- Two syringe pumps (Fusion 4000-X and Fusion 6000-X, Chemyx);
- Two syringes: one stainless steel syringe (20 mL) for the shell component and one plastic syringe (50 mL) for the core component;
- A coaxial nozzle (inner: 22G; outer: 18G, Ramehart). Alternative nozzle sizes can be used depending on the desired filament diameter and morphology;
- A stainless-steel coagulation bath (~81 cm in length), selected for its chemical resistance to a wide range of solvents;
- A custom-built submersible conveyor belt system;
- A custom-built filament collection winder;
- An infrared (IR) ceramic heater (1500 W, Brightown).

The coagulation bath is mounted on a T-slot aluminum frame equipped with adjustable feet and rails. The rail allows the collection winder to be repositioned along the length of the bath, enabling precise control of the filament dwell time without altering the extrusion or collection speeds. The height of the winder is also adjustable via a dual-knob bracket system. To reduce filament adhesion and the rotational inertia of the winder, the collection rods were fabricated from PTFE, allowing the motor to operate at a lower torque.

The conveyor belt is tensioned within a compact aluminum frame designed to sit inside the coagulation bath. A variable-speed motor located above the bath transmits motion to the belt via a timing belt connected to a common shaft. This motor placement avoids the need for submersible motors and allows the belt to maintain a low-profile geometry within the bath.

The setup is compatible with both wet and dry-jet wet spinning configurations. In the dry-jet wet spinning mode (used in this work), the extrusion nozzle is positioned slightly above the liquid level of the coagulation bath, creating an air gap. For standard wet spinning, the needle can be inserted into a designated port located ~5 cm below the top of the bath rim.

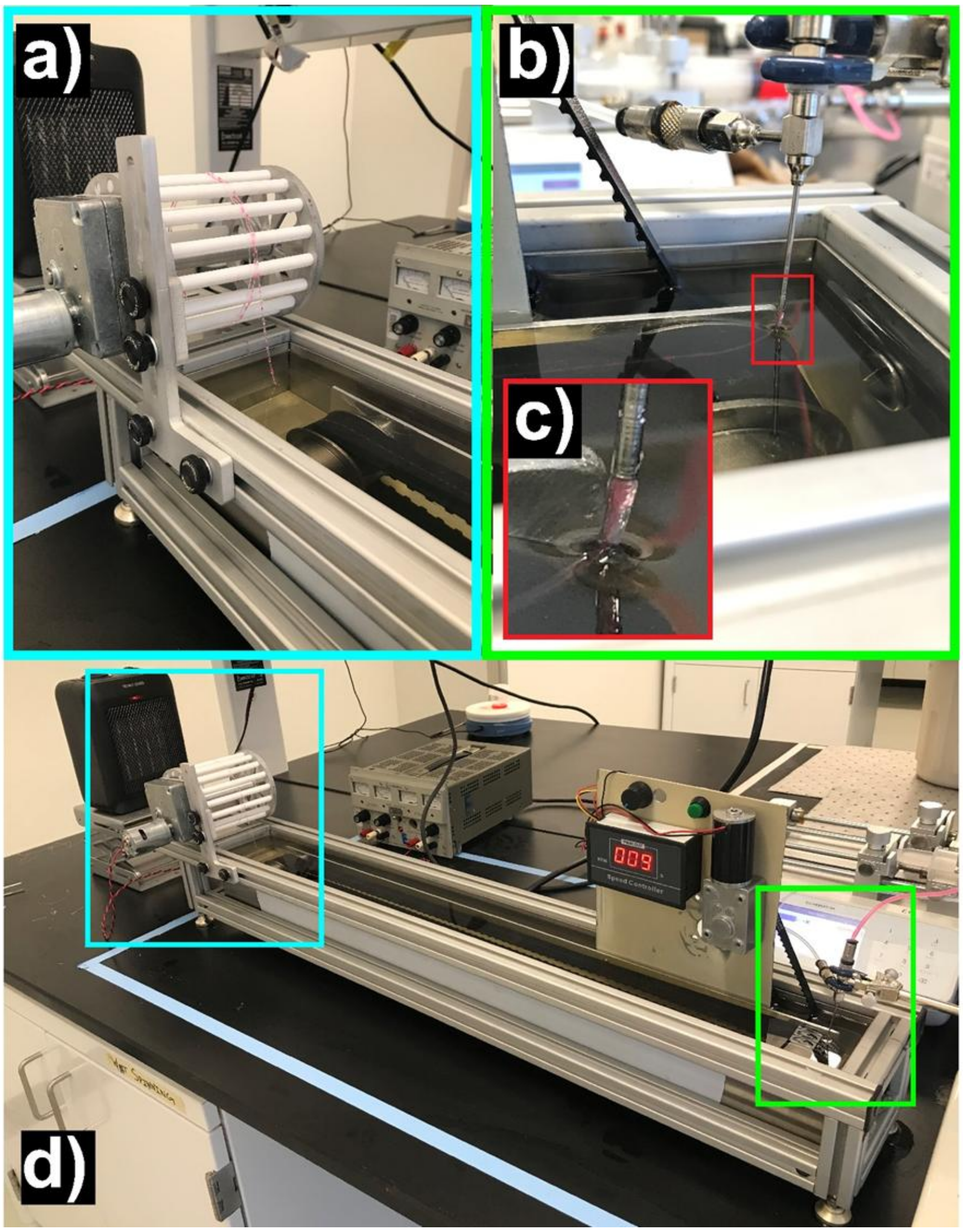

Figure S2. Photographs of the fabricated wet spinning apparatus in operation: a) winder collecting filament from coagulation bath (blue inset); b) coaxial needle extruding onto the submerged conveyor belt (green inset) with c) zoomed-in view of the needle tip positioned above the liquid level, showing the air gap (red inset), and d) overview of the complete filament spinning apparatus, including the conveyor belt placed inside the coagulation bath.

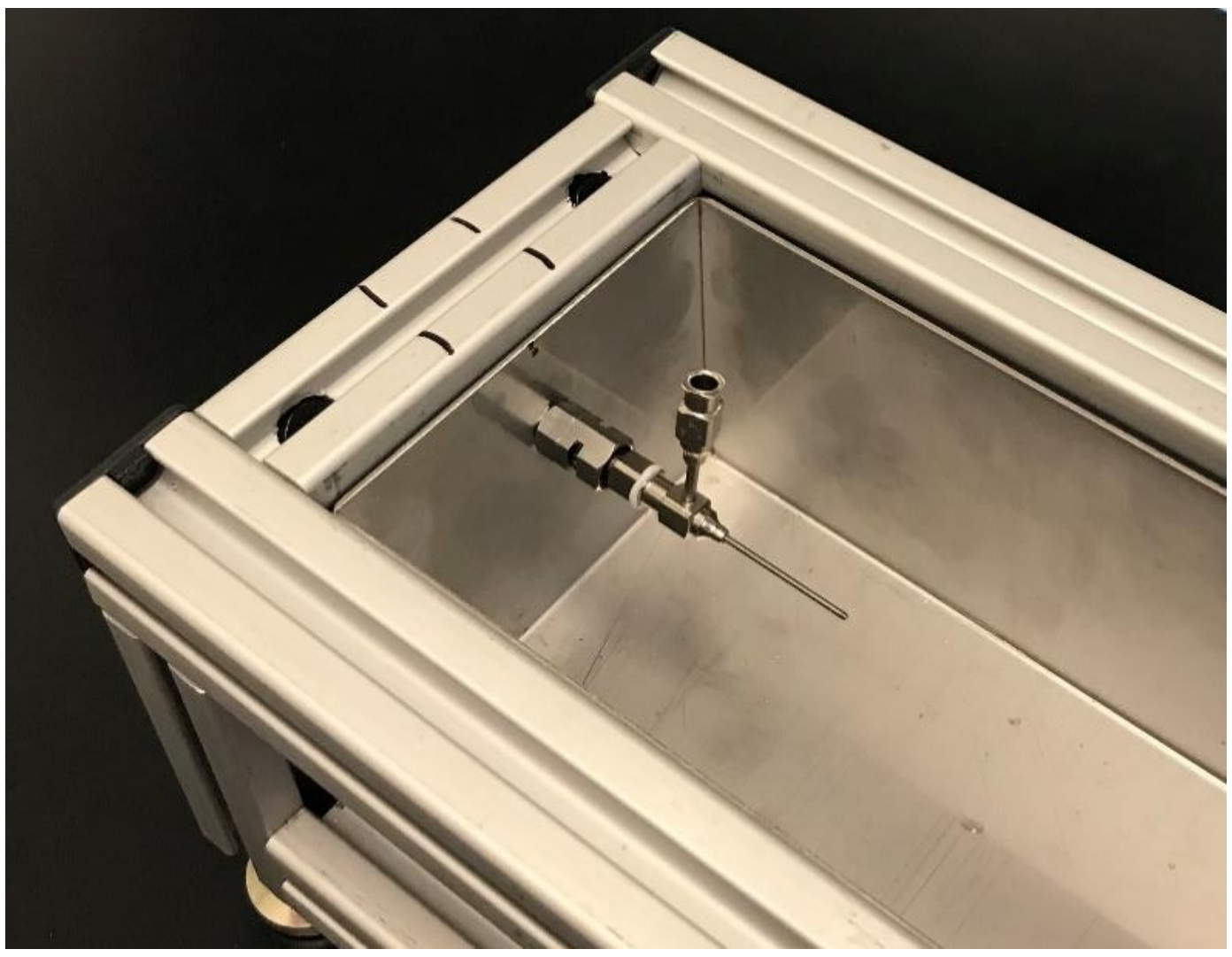

Figure S3. Photograph of the coaxial needle configured for standard wet spinning. The needle is affixed to the side wall of the stainless-steel coagulation bath, positioned horizontally to allow filament extrusion parallel to the bath floor.

S3. Rheological data analysis

For comparison with the flow curves, the apparent wall shear rate in the inner needle during core extrusion was estimated as Eq S1.

$$\dot{\gamma}_{app} = \frac{32Q}{\pi D^3} \quad \text{(Eq S1)}$$

Where Q is the volumetric flow rate and D is the inner needle diameter. Using Q = 0.1 mL min^{-1} and D = 0.406 mm, an apparent shear rate of 254 s^{-1} was obtained. This value was used to estimate the shear conditions experienced by the core during extrusion.

Table S1. Rheological parameters of the shell and core formulations.

Formulation	K (Pa s^n)	n	R^2	η at 254 s^{-1} (Pa s)	G'$_{LVR}$ (Pa)	G'-G" crossover (%)
MC-CNC hydrogel	28.0	0.050	0.986	0.1155	~203	~94
Pickering emulsion	0.236	0.235	0.993	0.00367	~2.00	~21
Corn oil	0.0601	1.01	N/A	0.0635	-	-

* Corn oil was not included in the oscillatory amplitude sweep analysis.

S4. Mechanical properties of shell-only MC-CNC filaments

Uniaxial tensile tests were performed on individual shell-only MC-CNC filaments using a dynamic mechanical analyzer (DMA 850, TA Instruments) with a gauge length of 1 cm and a displacement rate of 0.25 mm min^{-1}. Prior to testing, the filaments were dried at 105 °C and stored in a desiccator to minimize moisture effects. Filament diameters measured by optical microscopy were used to calculate cross-sectional area, considering the filaments as cylinders. Young's modulus was determined from the slope of the initial linear region of the stress-strain curves. Nine individual filaments were included in the analyses and a representative curve is shown in Figure S4.

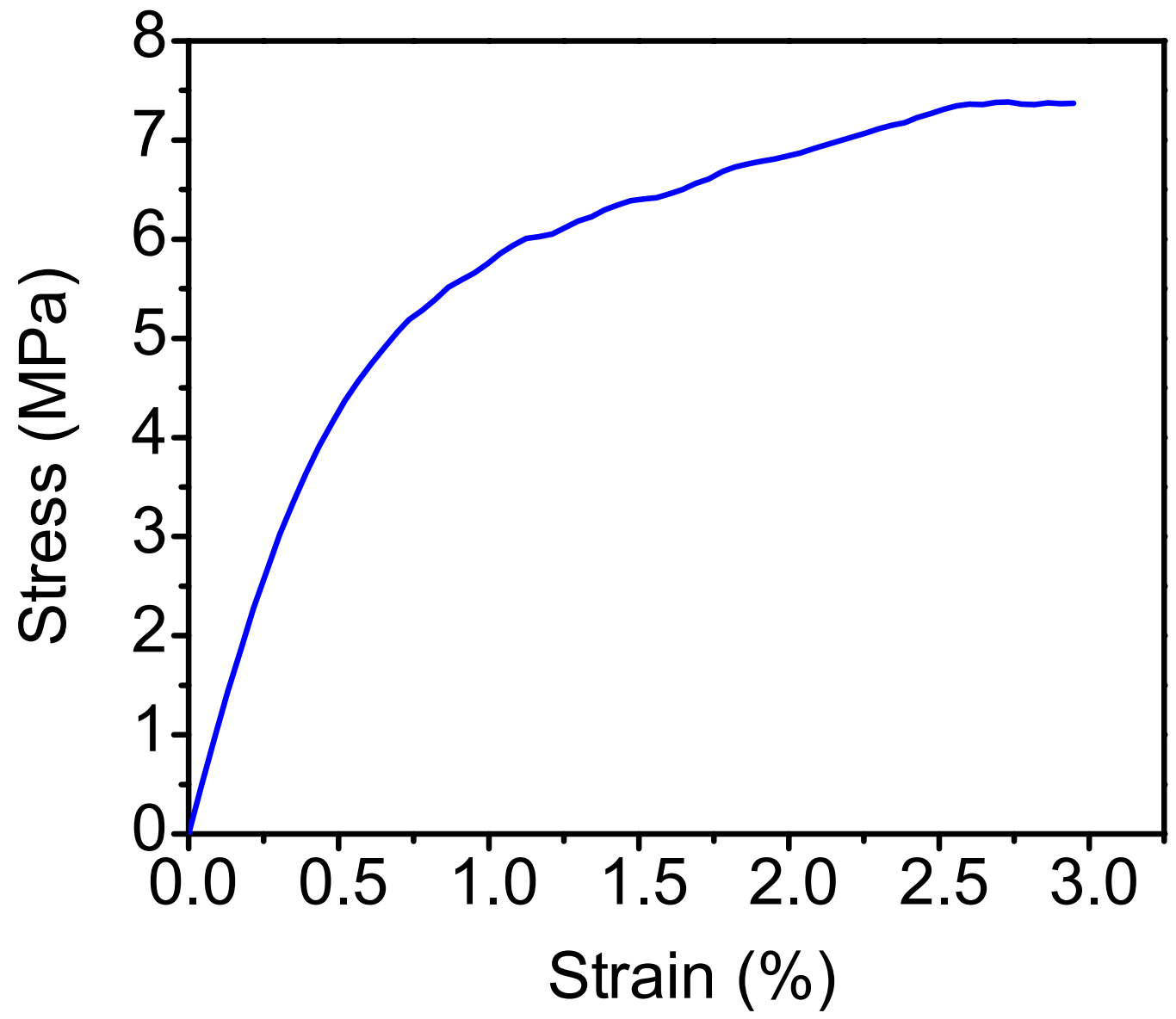

Figure S4. Representative stress–strain curve of a shell-only MC-CNC filament obtained by uniaxial tensile testing.

S5. Additional Pickering emulsion characterization

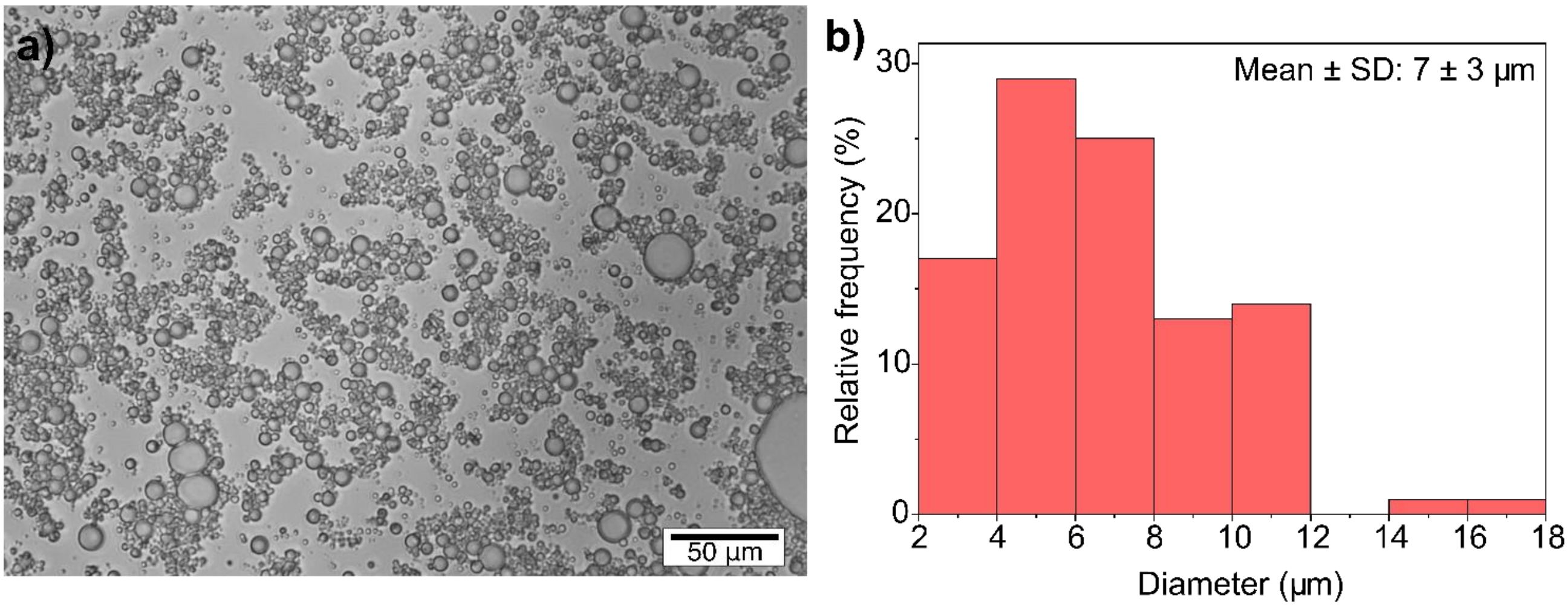

Figure S5. a) Optical microscopy of a CNC-stabilized corn-oil-in-water Pickering emulsion after extrusion through the coaxial nozzle and b) oil droplet diameter distribution histogram for comparison with starting emulsion shown in Figure 4.

S6. Additional oil-filled filament characterization

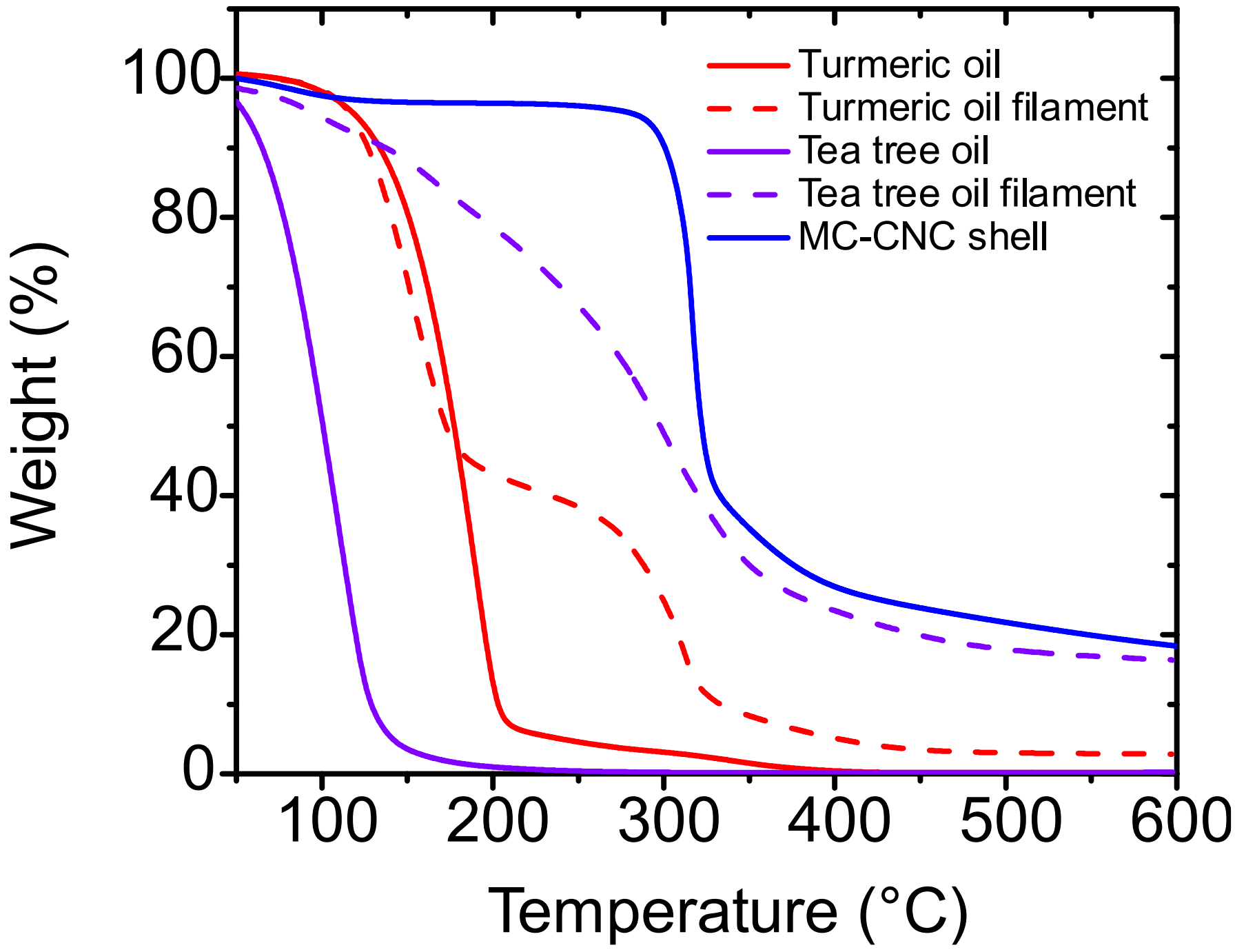

Figure S6. Thermogravimetric analysis (TGA) curves of turmeric and tea tree oils, and their corresponding oil-filled filaments, used to estimate oil content based on thermal degradation profiles.

Table S2. Summary of estimated oil content determined for the prepared filaments, including values expressed as weight percentage (wt%) and mass of oil per unit length (mg cm^{-1}). Oil content was estimated via TGA.

Internal component	Estimated oil content	
	**wt%**	**mg cm^{-1}**
Tea tree oil	5.2	0.02
Turmeric oil	35	0.3
Corn oil	47	0.4
CNC-stabilized emulsion of corn oil	24	0.1

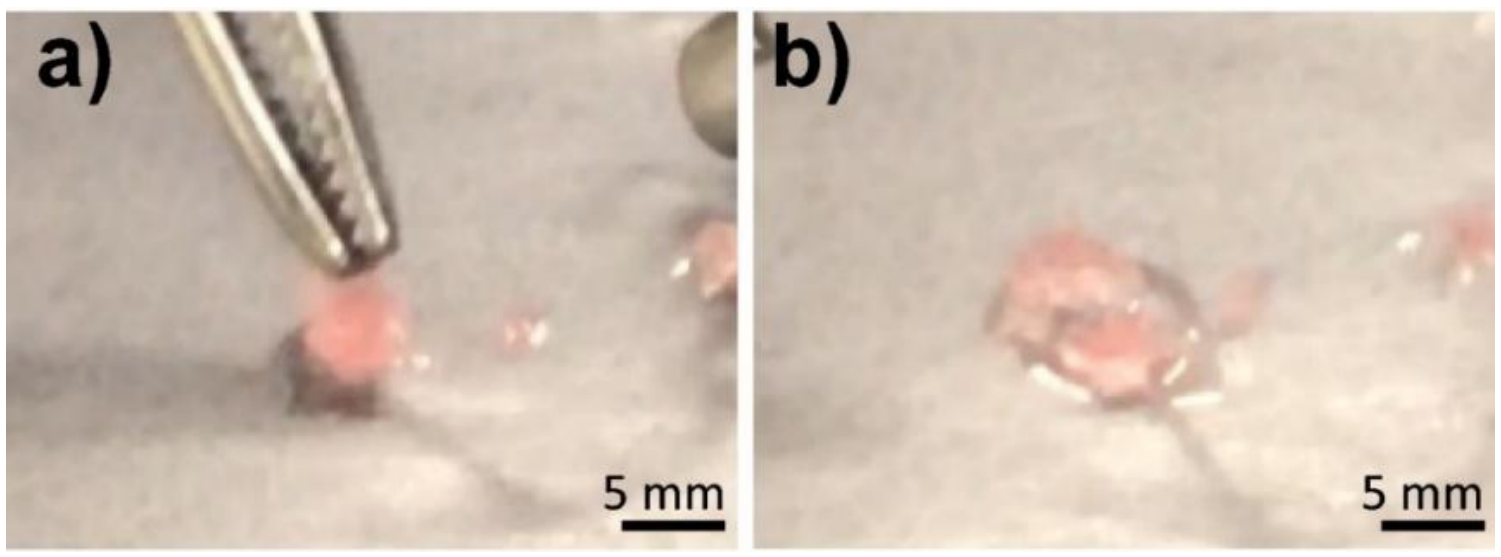

Figure S7. Demonstration of mechanical disruption of bead-on-string filament: a) application of pressure to an oil-filled region, and b) subsequent release of the encapsulated oil upon rupture. The oil was dyed using Nile red.
